# Night work and cancer risk: results from the ELSA-Brasil worker cohort

**DOI:** 10.11606/s1518-8787.2026060007579

**Published:** 2026-08-26

**Authors:** Débora Cristina de Almeida Mariano Bernardino, Ubirani Barros Otero, Isiyara Taverna Pimenta, Rosane Harter Griep, Luana Giatti, Sandhi Maria Barreto, Maria de Jesus Mendes da Fonseca

**Affiliations:** I Instituto Nacional de Câncer Coordenação de Prevenção e Vigilância Área Técnica Ambiente Trabalho e Câncer Rio de Janeiro RJ Brasil Instituto Nacional de Câncer. Coordenação de Prevenção e Vigilância. Área Técnica Ambiente Trabalho e Câncer. Rio de Janeiro, RJ, Brasil; II Fundação Oswaldo Cruz Escola Nacional de Saúde Pública Rio de Janeiro RJ Brasil Fundação Oswaldo Cruz. Escola Nacional de Saúde Pública. Rio de Janeiro, RJ, Brasil; III Fundação Oswaldo Cruz Instituto Leônidas e Maria Deane Manaus AM Brasil Fundação Oswaldo Cruz. Instituto Leônidas e Maria Deane. Manaus, AM, Brasil; IV Fundação Oswaldo Cruz Laboratório de Educação em Saúde e Meio Ambiente Rio de Janeiro RJ Brasil Fundação Oswaldo Cruz. Laboratório de Educação em Saúde e Meio Ambiente. Rio de Janeiro, RJ, Brasil; V Universidade Federal de Minas Gerais Programa de Pós-graduação em Saúde Pública Belo Horizonte MG Brasil Universidade Federal de Minas Gerais. Programa de Pós-graduação em Saúde Pública. Belo Horizonte, MG, Brasil; VI Universidade Federal de Minas Gerais Faculdade de Medicina Departamento de Medicina Preventiva e Social Belo Horizonte MG Brasil Universidade Federal de Minas Gerais. Faculdade de Medicina. Departamento de Medicina Preventiva e Social. Hospital das Clínicas. Belo Horizonte, MG, Brasil

**Keywords:** Night Work, Occupational Exposure, Occupational Cancer, Cancer, Cohort Studies

## Abstract

**OBJECTIVE:**

To estimate the association between exposure to night work and the incidence of breast, prostate, and colorectal cancer among participants in the Estudo Longitudinal de Saúde do Adulto (ELSA-Brasil – Longitudinal Study of Adult Health).

**METHODS:**

ELSA-Brasil is a multicenter cohort study conducted in the Northeast, South, and Southeast regions of Brazil. A total of 14,787 participants were included at baseline (2008–2010) and followed through 2023, with a mean follow-up duration of 13 years (SD = 2.22). Exposure to night work was defined based on self-reported occupational history, considering cumulative duration. Incidence cases of cancer were identified during follow-up. Associations were estimated using Cox regression models, adjusted for sociodemographic, behavioral, and occupational variables.

**RESULTS:**

Exposure to night work for more than ten years was associated with an increased risk of prostate cancer (HR = 1.38; 95% CI 1.09–1.75) and colorectal cancer (HR = 1.35; 95% CI 1.09–1.68), compared with exposure to day work. No association was observed between breast cancer and night work (HR = 0.89; 95% CI 0.65–1.22).

**CONCLUSION:**

The results suggest that prolonged exposure to night work may increase the risk of cancer, particularly prostate and colorectal cancer. This is the first study from the ELSA-Brasil cohort to analyze cancer incidence, expanding knowledge on work-related cancer and reinforcing the carcinogenic potential of night work, with implications for occupational health surveillance and cancer prevention policies in public health.

## INTRODUCTION

The International Agency for Research on Cancer has classified night work as a probable occupational carcinogen for humans, associated with breast, prostate, and colorectal cancers^1^. This classification is based on limited evidence of carcinogenicity in humans, combined with sufficient evidence from experimental studies and strong mechanistic support, which point to the disruption of the circadian rhythm as a biologically plausible mechanism for carcinogenesis^2,3^. However, epidemiological studies investigating the association between night work and cancer still present heterogeneous and inconsistent results.

Hong et al.^4^ reported an increased risk of breast cancer in women exposed to night work, while Lorca et al.^5^ observed an increased risk of prostate cancer in men. In contrast, Rivera-Izquierdo et al.^6^ and Sweeney et al.^7^ did not confirm these associations. For colorectal cancer, there are reports of an increased risk, particularly among workers on long-duration night shifts^8^, although this association has not been observed in other studies^9^.

In Brazil, studies on work-related cancer remain scarce, especially longitudinal investigations into night work and cancer risk. In part, this limitation stems from the lack of consistent occupational information in national cancer registries, making it difficult to measure occupational exposures^10^. Furthermore, international epidemiological literature still has limitations related to heterogeneity in the definition of night work, the cumulative measurement of exposure, temporal relationships, and dose-response analyses. To date, no Brazilian prospective studies on night work and cancer risk have been identified. In this context, the Estudo Longitudinal de Saúde do Adulto (ELSA-Brasil – Longitudinal Study of Adult Health) constitutes a strategic resource for investigating this association, due to its prospective design and the availability of detailed information on working conditions and health. Additionally, the characterization of exposure to night work throughout one’s working life allows for the exploration of aspects related to the timing and duration of exposure.

Thus, the objective of the study is to estimate the association between night work and the incidence of breast, prostate, and colorectal cancers in the ELSA-Brasil population, one of the largest cohorts of workers in Latin America.

## METHODS

### Study Design

ELSA-Brasil is a multicenter prospective cohort study initiated in 2008, comprising more than 15,000 active and retired civil servants, aged 35 to 74, from six Brazilian state capitals. The study investigates the incidence and risk factors for chronic diseases through medical examinations, quadrennial in-person interviews, and annual follow-up interviews (EAS). Methodological details have been previously described^11,12^. This longitudinal analysis used baseline data (2008–2010) and results on cancer incidence (2008–2023).

### Participants

The analysis included cancer-free participants from the ELSA-Brasil baseline cohort (2008–2010), whether employed or retired, with complete information on employment status, lifestyle habits, and socioeconomic characteristics. The Figure details the sample selection process, eligibility criteria, and losses that occurred during follow-up.

**Figure:**
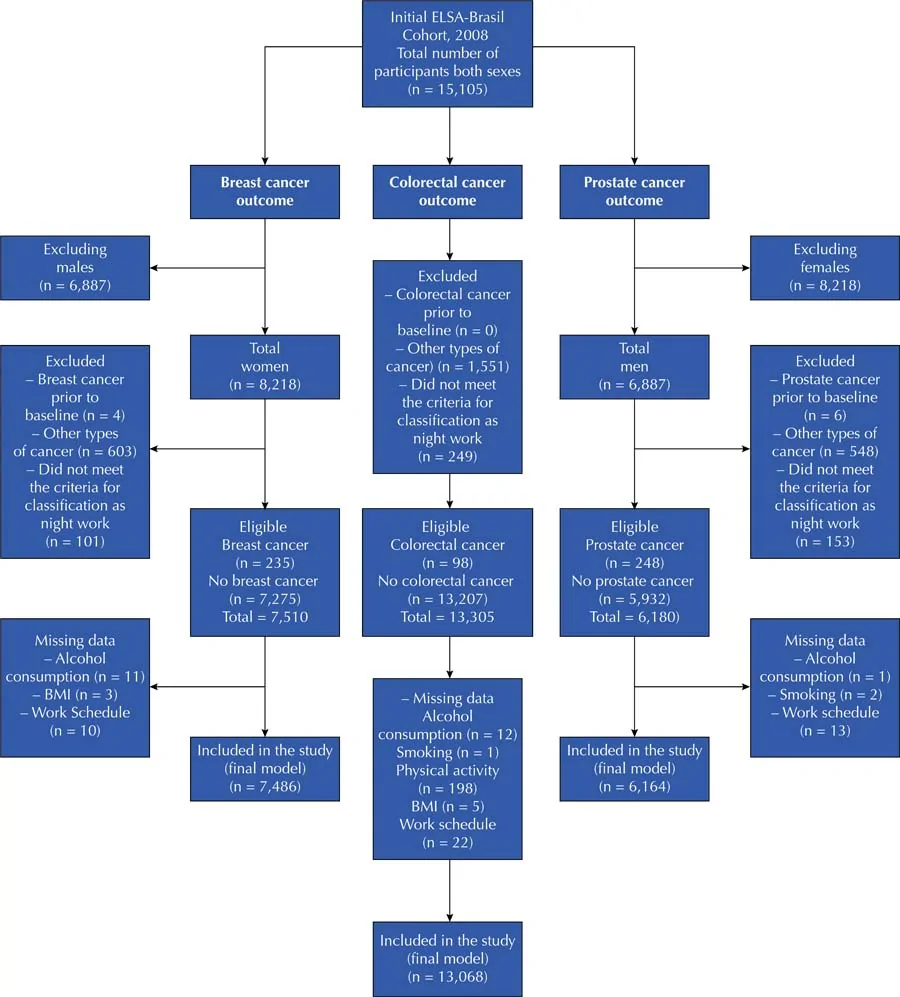
Flowchart of ELSA-Brasil participants (2008–2023) included in the study, for each of the outcomes of interest (breast, prostate, and colorectal cancer).

BMI: Body Mass Index; ELSA-Brasil: Estudo Longitudinal de Saúde do Adulto (Longitudinal Study of Adult Health).

### Outcome

Incident cases of breast, prostate, and colorectal cancer were identified during cohort follow-up (2008–2023) based on self-reported medical diagnoses in the EAS, including the anatomical location of the neoplasm. Cancer classification followed the guidelines of the International Agency for Research on Cancer and the main cancer classifications in Brazil^13^.

### Exposure

At baseline, exposure to shift work and night work was measured using the Occupational History questionnaire, with the following questions: “Considering all your jobs, do you currently work or have you ever worked on an on-call schedule?” (Yes, I currently work on an on-call schedule; Yes, I used to work on an on-call schedule but no longer do; No, never have); “In total, considering all your jobs, for how many years have you worked or have you worked on an on-call schedule?”; “Your most frequent on-call schedule is or was:” (Day shifts only; Night shifts only; Mixed); “Please describe what your most frequent on-call schedule is or was:” (12/36-hour shifts; 12/60-hour shifts; One 24-hour shift per week; One 12-hour shift per week; Two 12-hour shifts per week; Other, please specify).

Based on the collected data, the exposure variable was defined by considering the work schedule and current and past exposure to night work. Classification as a night worker followed the following criteria: individuals who work or have worked night shifts for at least 8 consecutive hours between 10:00 p.m. and 5:00 a.m., at least four times a month. The duration of exposure to night work, measured in years, was also considered. The definition adopted was based on a previous ELSA-Brasil study^14^ and aimed to prioritize the identification of habitual night work exposure throughout the occupational lifespan, thereby increasing the specificity of the measurement and reducing potential classification bias. To assess the duration of night work, participants were categorized into ≤ 10 years and > 10 years, with the aim of distinguishing longer exposures that are potentially associated with the cumulative effect of circadian dysregulation, given the lack of consensus in the literature regarding the ideal cutoff point for assessing this exposure in cancer studies.

### Covariates

The covariates included in the study were selected based on their carcinogenic potential and epidemiological relevance to the types of cancer investigated. Behavioral variables recognized in the literature as risk factors for cancer were: excessive alcohol consumption (consumption > 210 g/week for men and > 140 g/week for women); smoking (never smoked, former smoker, current smoker); leisure-time physical activity (low; moderate/vigorous, according to the long version of the International Physical Activity Questionnaire – IPAQ) ^15^; nutritional status assessed by Body Mass Index (BMI) (underweight/normal weight < 18 kg/m^2^ to ≤ 24.9 kg/m^2^; overweight ≥ 25 kg/m^2^ to > 40 kg/m^2^); processed meat consumption (no/yes); and red meat consumption (≤ 71 g/day; > 71 g/day)^16^. Sociodemographic variables that may contribute to the outcome of interest included: age (continuous variable; also categorized as 35–50 years and > 50 years, due to the increased risk of cancer after age 50 in the incidence analysis)^17^; biological sex (male/female); and educational attainment (elementary, high school, college, or higher), chosen in lieu of income. This decision is based on the fact that, although both are correlated socioeconomic indicators, educational attainment exhibits greater temporal stability, is less susceptible to measurement error, and constitutes a structural determinant of occupational trajectory. Thus, its inclusion as a marker of socioeconomic status is conceptually more appropriate in the context of this study, in accordance with methodological recommendations for multivariate epidemiological designs.

### Ethical Considerations

ELSA-Brasil was approved by the Ethics Committees of the participating institutions and by the Comissão Nacional de Ética em Pesquisa (CONEP – National Research Ethics Commission). This study was approved on January 26, 2023, by the Research Ethics Committee of the Escola Nacional de Saúde Pública/Fundação Oswaldo Cruz (ENSP/Fiocruz), under approval number 66407322.3.0000.5240.

### Statistical Analysis

Analyses were performed separately for each type of primary cancer (breast, prostate, and colorectal), considering gender-specific factors. Incidence rates (in person-years) were calculated based on the time elapsed between the start of follow-up and the date of diagnosis. Participants who did not develop the cancers of interest or who died during the follow-up period were treated as censored.

The association between night work and cancer incidence was estimated using Cox regression models, with calculation of hazard ratios (HR) and 95% confidence intervals (95% CI). Adjustment variables were selected based on the scientific literature and the recommendations of the International Agency for Research on Cancer, prioritizing risk factors recognized as carcinogenic for the cancer types studied. Covariates were included in the models incrementally, also considering statistical significance in the univariate analyses (p < 0.10).

For each of the cancer types investigated, simple models were obtained (Model A). The final model for breast cancer was adjusted for excessive alcohol consumption, smoking, BMI, and age (Model B). For prostate cancer, the final model was adjusted for excessive alcohol consumption, smoking, and age (Model D). The final model for colorectal cancer was adjusted for alcohol consumption, smoking, processed meat consumption, physical activity, BMI, age, and sex (Model E). Additionally, a complementary model was adjusted by including the variable “educational level” to compare the models and verify whether overfitting occurred because of its incorporation into the final model (Model C).

Interaction tests between night work and educational attainment did not indicate any change in effect. The assumption of proportional hazards was assessed using Schoenfeld residual plots, with no evidence of violation.

The analyses were performed using R software (version 4.4.0), with the survival, ggsurvfit, ggplot2, ggfortify, survminer, car, and DescTools packages.

## RESULTS

In the ELSA-Brasil baseline cohort (2008–2010), after excluding participants with a prior cancer diagnosis, 14,787 individuals were included, of whom 81.0% worked daytime shifts. Most of the study population consisted of women (54.7%). A higher proportion of night shift work was observed among men. Workers with higher levels of education and income were concentrated in the day shift, while those with lower levels of education and income were more frequently found in the night shift. Among lifestyle factors, a higher prevalence of excessive alcohol consumption, overweight status, and lower levels of physical activity was observed among night shift workers (Table 1).

**Table 1:** Distribution of characteristics of the study population by work shift – ELSA-Brasil, 2008–2010 baseline. (n = 14,787)

Category	Work Schedule			Total n (%)	p-value
	Day shift worker n = 11,973 n (%)	Night shift worker (≤ 10 years) n = 1,541 n (%)	Night shift worker (> 10 years) n = 1,273 n (%)		
Gender					
Male	5,277 (78.8)	854 (12.8)	565 (8.4)	6,696 (45.3)	< 0.0001
Female	6,696 (82.8)	687 (8.5)	708 (8.8)	8,091 (54.7)	
Age group					
35–50 years	5,607 (81.0)	736 (10.6)	578 (8.4)	6,921 (46.8)	0.4557
> 50 years	6,366 (80.9)	805 (10.2)	695 (8.8)	7,866 (53.2)	
Education					
Through elementary school	1,435 (76.7)	198 (10.6)	237 (12.7)	1,870 (12.6)	< 0.0001
High school	3,878 (75.7)	601 (11.7)	645 (12.6)	5,124 (34.7)	
Higher education or beyond	6,660 (85.5)	742 (9.5)	391 (5.0)	7,793 (52.7)	
*Per capita* household income					
Up to R$ 2,879.60	3,013 (76.9)	433 (11.1)	470 (12.0)	3,916 (26.6)	< 0.0001
From R$ 2,879.70 to R$ 5,344.70	3,574 (78.7)	494 (10.9)	473 (10.4)	4,541 (30.8)	
≥ R$ 5,344.80	5,341 (85.1)	612 (9.7)	326 (5.2)	6,279 (42.6)	
Excessive drinking					
No	11,088 (81.1)	1,397 (10.2)	1,193 (8.7)	13,678 (92.6)	0.0062
Yes	876 (79.9)	142 (12.9)	79 (7.2)	1,097 (7.4)	
Smoker					
Never smoked	6,861 (81.4)	842 (10.0)	726 (8.6)	8,429 (57.0)	0.3262
Former smoker	3,568 (80.7)	478 (10.8)	377 (8.5)	4,423 (29.9)	
Current smoker	1,544 (79.8)	221 (11.4)	169 (8.7)	1,934 (13.1)	
Processed meat consumption					
No	1,347 (78.9)	182 (10.7)	178 (10.4)	1,707 (11.5)	0.0140
Yes	10,626 (81.2)	1,359 (10.4)	1,095 (8.4)	13,080 (88.5)	
Red meat consumption					
Moderate (≤ 71 g/day)	6,761 (82.5)	793 (9.7)	644 (7.9)	8,198 (55.4)	< 0.0001
Excessive (> 71 g/day)	5,212 (79.1)	748 (11.4)	629 (9.5)	6,589 (44.6)	
BMI					
Underweight/normal weight	4,512 (82.8)	542 (9.9)	394 (7.2)	5,448 (36.9)	< 0.0001
Overweight	7,456 (79.9)	998 (10.7)	879 (9.4)	9,333 (63.1)	
Physical activity					
Moderate/vigorous	2,757 (81.7)	379 (11.2)	237 (7.0)	3,373 (23.1)	0.0006
Weak	9,057 (80.8)	1,138 (10.2)	1,008 (9.0)	11,203 (76.9)	

BMI: Body Mass Index.

After a mean follow-up of 13 years (SD = 2.22 years), 539 participants (3.6%) developed the types of cancer of interest in the study. Among women, 239 (3.1%) were diagnosed with breast cancer; among men, 252 (4.0%) developed prostate cancer. Colorectal cancer was identified in 101 participants (0.7%), including 51 men and 50 women. The incidence rates for breast and prostate cancers were 2.34 cases (95% CI 2.05–2.66) and 3.05 (95% CI 2.69–3.46) per 1,000 person-years, respectively. For colorectal cancer, the incidence rate was 5.63 (95% CI 4.59–6.84) per 10,000 person-years. Higher incidence rates were observed among individuals with excessive alcohol consumption for all three types of cancer analyzed, with the highest rate observed for colorectal cancer (8.67 cases per 10,000 person-years). For this same cancer type, higher rates were also observed among those who consumed processed meat (5.72/10,000), were overweight (6.03/10,000), or reported low levels of physical activity (5.79/10,000), compared with the respective reference groups (Table 2).

**Table 2:** Incidence of breast, prostate, and colorectal cancer according to sociodemographic, behavioral, and occupational characteristics among ELSA-Brasil participants, 2008–2023.

Variable	Breast Cancer				Prostate cancer				Colorectal cancer			
	n	Cases	Person-year	Incidence rate^a^ 95% CI	n	Cases	Person-year	Incidence rate^a^ 95% CI	n	Cases	Person-year	Incidence rate^b^ 95% CI
Sex												
Male	-	-	-	-	6,333	252	82,496.8	3.05 (2.69–3.46)	6,132	51	79,819.8	6.39 (4.76–8.40)
Female	7,611	239	102,133.8	2.34 (2.05–2.66)	-	-	-	-	7,422	50	99,580.6	5.02 (3.73–6.62)
Age group												
35–50 years	3,613	103	49,402.5	2.08 (1.70–2.53)	3,076	36	41,344.7	0.87 (0.61–1.21)	6,575	25	89,212.8	2.80 (1.81–4.14)
> 50 years	3,998	136	52,731.4	2.58 (2.16–3.05)	3,257	216	41,152.1	5.25 (4.57–6.00)	6,979	76	90,187.6	8.43 (6.64–10.55)
Education												
Elementary school or less	737	16	9,396.5	1.70 (0.97–2.77)	1,075	37	13,316.9	2.78 (1.96–3.83)	1,770	11	22,155.5	4.96 (2.48–8.88)
Median	2,798	61	37,707.1	1.62 (1.24–2.08)	2,144	52	28,134.8	1.85 (1.38–2.42)	4,861	32	64,725.6	4.94 (3.38–6.98)
Higher or more	4,076	162	55,030.2	2.94 (2.51–3.43)	3,114	163	41,045.1	3.97 (3.38–4.63)	6,923	58	92,519.4	6.27 (4.76–8.10)
Income												
Up to R$ 2,489.00	2,066	36	27,533.4	1.31 (0.92–1.81)	1,727	55	22,258.8	2.47 (1.86–3.22)	3,723	21	48,847.7	4.30 (2.66–6.57)
From R$ 2,490.00 to R$ 4,979.00	2,482	74	33,423.9	2.21 (1.74–2.78)	1,869	47	24,393.6	1.93 (1.42–2.56)	4,260	30	56,556.4	5.30 (3.58–7.57)
≥ R$ 4,980.00	3,027	129	40,765.3	3.16 (2.64–3.76)	2,708	148	35,523.8	4.17 (3.52–4.89)	5,508	50	73,291.5	6.82 (5.06–8.99)
Excessive alcohol consumption												
No	7,330	225	98,463.0	2.29 (2.00–2.60)	5,565	220	72,765.8	3.02 (2.64–3.45)	12,540	90	166,468.3	5.41 (4.35–6.65)
Yes	263	14	3,485.8	4.02 (2.20–6.74)	760	32	9,674.6	3.31 (2.26–4.67)	988	11	12,690.7	8.67 (4.33–15.51)
Smoking												
Never smoked	4,753	155	64,140.0	2.42 (2.05–2.83)	3,211	118	42,522.8	2.77 (2.30–3.32)	7,742	51	103,659.0	4.92 (3.66–6.47)
Former smoker	1,923	61	25,678.4	2.38 (1.82–3.05)	2,224	107	28,745.1	3.72 (3.05–4.50)	4,011	32	52,598.6	6.08 (4.16–8.59)
Smoker	935	23	12,315.5	1.87 (1.18–2.80)	897	27	11,214.8	2.41 (1.59–3.50)	7,742	51	103,659.0	4.92 (3.66–6.47)
Processed meat consumption												
No	996	29	13,047.3	2.22 (1.49–3.19)	625	37	7,939.4	4.66 (3.28–6.42)	1,565	10	20,237.6	4.94 (2.37–9.09)
Yes	6,615	210	89,086.5	2.36 (2.05–2.70)	5,708	215	74,557.5	2.88 (2.51–3.30)	11,989	91	159,162.9	5.72 (4.60–7.02)
Red meat consumption												
≤ 71 g/day	4,820	167	64,630.9	2.58 (2.21–3.01)	2,885	119	37,485.4	3.17 (2.63–3.80)	7,470	51	98,970.2	5.15 (3.84–6.78)
> 71 g/day	2,791	72	37,503.0	1.92 (1.50–2.42)	3,448	133	45,011.4	2.95 (2.47–3.50)	6,084	50	80,430.3	6.22 (4.61–8.20)
BMI												
Underweight/normal weight	2,997	89	40,424.6	2.20 (1.77–2.71)	2,152	92	28,202.6	3.26 (2.63–4.00)	5,001	33	66,611.0	4.95 (3.41–6.96)
Overweight	4,611	150	61,674.9	2.43 (2.06–2.85)	4,179	160	54,266.5	2.95 (2.51–3.44)	8,548	68	112,727.4	6.03 (4.68–7.65)
Physical activity												
Moderate/vigorous	1,478	50	19,940.6	2.51 (1.86–3.31)	1,649	90	21,675.6	4.15 (3.34–5.10)	3,009	22	40,050.0	5.49 (3.44–8.32)
Poor	6,019	189	80,579.6	2.35 (2.02–2.70)	4,592	159	59,505.8	2.67 (2.27–3.12)	10,342	79	136,465.9	5.79 (4.58–7.21)
Work schedule												
Day shift worker	6,193	200	83,045.0	2.41 (2.09–2.77)	4,856	197	63,370.8	3.11 (2.69–3.57)	10,729	77	142,135.4	5.42 (4.28–6.77)
Night shift worker ≤ 10 years	642	24	8,600.2	2.79 (1.79–4.15)	778	25	10,142.5	2.46 (1.60–3.64)	1,385	14	18,273.3	7.66 (4.19–12.85)
Night shift worker >10 years	665	11	9,044.8	1.22 (0.61–2.18)	533	25	6,842.2	3.65 (2.36–5.39)	1,169	7	15,484.6	4.52 (1.82–9.31)

95% CI: 95% confidence interval.

^a^ Event rates per 1,000 person-year.

^b^ Event rates per 10,000 person-year.

Considering night work and the three types of cancer evaluated, no association was observed with breast cancer among women (HR = 0.89; 95% CI 0.65–1.22). Regarding prostate cancer, after adjusting for alcohol consumption, smoking, and age, men exposed to night work for more than 10 years had a 38% increased risk of this type of cancer (95% CI 1.09–1.75). Exposure to night work for more than 10 years, after adjusting for alcohol consumption, smoking, processed meat, leisure-time physical activity, BMI, age, and sex, was associated with an increased risk of developing colorectal cancer (HR = 1.35; 95% CI 1.09–1.68). However, the strength of this association, for both prostate and colorectal cancer, was attenuated after adjusting by educational level (Table 3).

**Table 3:** Risk of breast, prostate, and colorectal cancer according to exposure to night work among participants in the ELSA-Brasil 2008–2023 study.

Exposure category	Breast cancer			Prostate cancer			Colorectal cancer		
	HR (95% CI)^a^	HR (95% CI)^b^	HR (95% CI)^c^	HR (95% CI)^a^	HR (95% CI)^d^	HR (95% CI)^c^	HR (95% CI)^a^	HR (95% CI)^e^	HR (95% CI)^c^
Day shift worker (Reference)	1.00	1.00	1.00	1.00	1.00	1.00	1.00	1.00	1.00
Night shift worker ≤ 10 years	0.98 (0.72–1.34)	1.00 (0.73–1.37)	1.00 (0.73–1.37)	1.00 (0.79–1.26)	1.05 (0.83–1.32)	0.99 (0.78–1.24)	1.13 (0.91–1.41)	1.07 (0.86–1.34)	0.99 (0.79–1.24)
Night shift worker >10 years	0.92 (0.67–1.25)	0.89 (0.65–1.22)	0.87 (0.64–1.20)	1.41 (1.12–1.79)	1.38 (1.09–1.75)	1.24 (0.98–1.57)	1.38 (1.11–1.71)	1.35 (1.09–1.68)	1.15 (0.92–1.43)

HR: hazard ratio; 95% CI: 95% confidence interval.

^a^ Unadjusted Cox regression model.

^b^ Cox regression model adjusted for: alcohol consumption, smoking, BMI, and age.

^c^ Additional adjustment for educational level.

^d^ Cox regression model adjusted for: alcohol consumption, smoking, and age.

^e^ Cox regression model adjusted for: alcohol consumption, smoking, processed meat, physical activity, BMI, sex, and age.

## DISCUSSION

The objective of this study was to estimate the association between night work and the incidence of breast, prostate, and colorectal cancers in the ELSA-Brasil population. In this large cohort of Brazilian civil servants, exposure to night work for more than 10 years was associated with an increased risk of prostate and colorectal cancer compared with day workers and those with shorter exposure durations.

Our results are consistent with evidence from cohorts in the United States, involving women^18^, and in Norway, involving men^19^, which identified a 64% increase in the risk of colorectal cancer and an 86% increase in the risk of prostate cancer, respectively. Both studies considered night shift work duration of 10 years or more. A common and relevant feature shared by these studies and the present study is that the analyses considered lifetime exposure to night work, rather than just current exposure. This allowed for a more precise assessment of the cumulative effects of night work on cancer development through occupational history.

On the other hand, non-longitudinal studies^20^, with a mean follow-up of less than 10 years^21^, or those that measured exposure based on current work schedules at the start of follow-up^22^ reported no association between night work and the risk of colorectal and prostate cancer. We believe that the effect of night work on cancer development, which generally has a long latency period, especially for solid tumors, can be adequately assessed in long-term studies. Furthermore, it is essential to include a comprehensive review of workers’ occupational history, which allows for the accounting of past exposure, including its duration, thereby reducing the likelihood of underestimated risk estimates.

This finding is supported by authors such as Harma et al.^23^, who suggest that a short follow-up period and the lack of information on pre-baseline exposure limit the ability of cohort studies to assess the long-term health effects of night work. Shi et al.^8^ observed that the risk of colorectal cancer increased significantly with exposure to long-term rotating night shifts, especially among workers exposed for 15 years or more.

Additionally, Gustavsson et al.^24^ reported an increased risk of breast cancer (HR = 4.33; 95% CI 1.45–10.57) among women who worked night shifts for eight years or more in a Swedish cohort. However, the authors concluded that the limitations of their study—such as the short follow-up period, the small number of cases among workers with prolonged exposure to night work, and the lack of information on occupational history prior to baseline—compromised the accuracy of the estimates. These results underscore the importance of longitudinal studies among workers with prolonged exposure to assess the actual impact of night work exposure on the development of breast cancer.

Thus, our hypothesis is that the lack of association observed in the initial risk estimates for breast cancer in our Brazilian cohort (ELSA-Brasil) may be related to the need for a longer follow-up period, which would include a larger number of exposed workers, as well as the long latency period characteristic of this cancer.

Regarding the attenuation of the estimates after adjusting the prostate and colorectal cancer models for educational attainment, we reaffirm that socioeconomic factors exert a strong influence on cancer incidence and mortality in the Brazilian context^25^. However, given the specific nature of this study, which aims to contribute to the understanding of the complex relationship between cancer and work in a cohort of Brazilian workers, it is not possible to analyze educational attainment in isolation as a socioeconomic factor that would act solely as a confounder, since it is part of the socio-occupational dimension that determines the probability of exposure to night work. In other words, exposure to night work depends on the type of occupation, which, in turn, is profoundly influenced by the individual’s educational level. In the present study, it was observed that 69.28% of workers exposed to night work for more than 10 years had completed high school or less (Table 1), indicating that prolonged exposure to this work schedule tends to occur among individuals with lower levels of education.

In this context, we hypothesize that more highly qualified workers tend to have greater autonomy to reorganize their professional trajectories and, whenever possible, transition to daytime shifts, thereby reducing their total exposure to night shifts. In contrast, those with lower levels of education, whose access to the labor market is typically more limited, often have fewer alternatives for changing their work schedules, which contributes to longer and more persistent exposure to night work. This dynamic is consistent with the literature describing the strong socioeconomic stratification in the distribution of working conditions and opportunities for occupational mobility^26^, reinforcing the need to consider educational attainment as an integral part of the structure that determines exposure itself, and not merely as a potential confounder.

Therefore, in the context of this study, including educational attainment in the final model would constitute overfitting. The epidemiological literature emphasizes that overfitting dilutes true associations, increases the standard error, and compromises causal interpretation^27^. In the case of exposure to night work, educational level is part of the structural correlates of exposure and, therefore, should not be automatically included in the models^27^. By controlling variables that make up the very structure generating the exposure, there is a risk of artificially reducing the magnitude of the effect and underestimating the true risk. Thus, we made a methodological choice not to adjust for educational attainment to preserve the internal validity of the estimates and avoid biases resulting from overadjustment, especially in a field where there is still a scarcity of national longitudinal studies capable of elucidating the relationship between night work and cancer incidence.

Considering this methodological rationale, the findings of this study gain greater consistency, since the absence of adjustment for variables structurally linked to exposure allows for a more reliable assessment of its potential effects. Our results point to an association between long-term night work and cancer, given that chronic exposure to this condition may promote the development of the disease through mechanisms such as the induction of inflammatory processes and the disruption of the circadian rhythm, corroborating the findings of other authors.

According to the International Agency for Research on Cancer^1^, exposure to artificial light during night shifts is the main cause of circadian rhythm disruption. Li et al.^3^ highlight that this phenomenon is considered a possible mechanism that promotes the development of cancer by impairing the control of cell cycles, reducing the efficiency of the immune response and DNA repair, with direct implications for cancer genesis. The authors emphasize that the association between exposure to night work and the development of endocrine cancers, such as breast, prostate, and colorectal cancers, is supported by strong mechanistic evidence and experimental studies in animals. These studies reveal the crucial role of circadian clock genes in regulating hormonal processes, as well as influencing enzyme secretion and the absorption of fluids and nutrients. Prolonged exposure to night work causes substantial changes in physiological rhythms, negatively impacting hormonal and metabolic balance, which may increase the risk of cancer^28,29^. Such evidence reinforces the importance of considering the impacts of circadian dysregulation on human health, especially among workers chronically exposed to night shifts. However, the relationships between night work and cancer risk in epidemiological studies have not yet been fully established. In this regard, our study helps to fill this gap, especially in the Brazilian context, where longitudinal studies capable of prospectively assessing the effects of night work on cancer incidence are scarce.

Finally, we highlight the significance of this study as the first worker cohort to estimate the risk of cancer associated with night work in Brazil, representing an unprecedented advance in the field of occupational health. The robustness of the ELSA-Brasil study, with its large sample size and detailed information on exposures, lends strength and originality to our study.

Despite these strengths, the findings should be interpreted in light of limitations inherent in occupational epidemiological studies. Of particular note is the possibility of the *“*healthy worker effect”—a phenomenon in which active workers tend to be in better health than the general population—which may contribute to an underestimation of the risk associated with night work. Furthermore, a longer follow-up period is needed, given the long latency period of solid tumors. The small number of exposed workers and, consequently, the low number of cases when considering the duration of exposure to night work may also limit the assessment of a possible risk gradient, which could more consistently demonstrate the association between duration of exposure and the development of the cancers investigated, especially breast cancer.

It is also worth noting that incident cancer cases were identified based on self-reported medical diagnoses in the health surveys, without validation by clinical records or medical reports, which may introduce classification bias. Furthermore, exposure to night work and lifestyle-related covariates were self-reported and measured only at baseline, which may reduce the accuracy of the measurements throughout the follow-up period and contribute to an underestimation of the observed associations.

## CONCLUSION

Our results suggest that prolonged exposure to habitual night work may be associated with an increased risk of cancer, particularly prostate and colorectal cancer. In this context, duration of exposure emerges as a relevant factor in assessing this risk, supporting the hypothesis of a possible cumulative effect and a dose-response relationship between night work and carcinogenesis. These findings expand our understanding of the influence of the work environment on the health of Brazilian public servants, confirming the carcinogenic potential of activities performed at night. The study reinforces the importance of discussing occupational risks and implementing preventive policies and public health interventions focused on night work.
