# Supplementary material for: Night work and cancer risk: results from the ELSA-Brasil worker cohort
Source: Rev Saude Publica. 2026 Aug 26;60:e40. doi: 10.11606/s1518-8787.2026060007579 (PMC13568599; doi:10.11606/s1518-8787.2026060007579)
Supplement: Translation

# Translation: Trabalho noturno e risco de câncer: resultados da coorte de trabalhadores ELSA-Brasil

**Authors:** Débora Cristina de Almeida Mariano Bernardino, Ubirani Barros Otero, Isiyara Taverna Pimenta, Rosane Harter Griep, Luana Giatti, Sandhi Maria Barreto, Maria de Jesus Mendes da Fonseca

**Affiliations:** I Brasil Instituto Nacional de Câncer. Coordenação de Prevenção e Vigilância. Área Técnica Ambiente Trabalho e Câncer. Rio de Janeiro, RJ, Brasil; II Brasil Fundação Oswaldo Cruz. Escola Nacional de Saúde Pública. Rio de Janeiro, RJ, Brasil; III Brasil Fundação Oswaldo Cruz. Instituto Leônidas e Maria Deane. Manaus, AM, Brasil; IV Brasil Fundação Oswaldo Cruz. Laboratório de Educação em Saúde e Meio Ambiente. Rio de Janeiro, RJ, Brasil; V Brasil Universidade Federal de Minas Gerais. Programa de Pós-graduação em Saúde Pública. Belo Horizonte, MG, Brasil; VI Brasil Universidade Federal de Minas Gerais. Faculdade de Medicina. Departamento de Medicina Preventiva e Social. Hospital das Clínicas. Belo Horizonte, MG, Brasil

**Keywords:** Trabalho Noturno, Exposição Ocupacional, Câncer Ocupacional, Câncer, Estudos de Coortes

## Abstract

**OBJETIVO:**

Estimar a associação entre a exposição ao trabalho noturno e a incidência de câncer de mama feminino, de próstata e de cólon-reto entre participantes do Estudo Longitudinal de Saúde do Adulto (ELSA-Brasil).

**MÉTODOS:**

O ELSA-Brasil é uma coorte multicêntrica, conduzida nas regiões Nordeste, Sul e Sudeste do Brasil. Foram incluídos 14.787 participantes da linha de base (2008–2010), acompanhados até 2023, com tempo médio de seguimento de 13 anos (DP = 2,22). A exposição ao trabalho noturno foi definida a partir da história ocupacional autorreferida, considerando a duração cumulativa. Casos incidentes de câncer foram identificados durante o seguimento. As associações foram estimadas por modelos de regressão de Cox, ajustados por variáveis sociodemográficas, comportamentais e ocupacionais.

**RESULTADOS:**

A exposição ao trabalho noturno por mais de dez anos foi associada a um maior risco de câncer de próstata (HR = 1,38; IC95% 1,09–1,75) e cólon-reto (HR = 1,35;IC95% 1,09–1,68), comparada à exposição ao trabalho diurno. Não foi observada associação entre o câncer de mama e o trabalho noturno (HR = 0,89; IC95% 0,65–1,22).

**CONCLUSÃO:**

Os resultados sugerem que a exposição prolongada ao trabalho noturno pode aumentar o risco de câncer, especialmente de próstata e cólon-reto. Este é o primeiro estudo do ELSA-Brasil a analisar a incidência de câncer, ampliando o conhecimento sobre o câncer relacionado ao trabalho e reforçando o potencial carcinogênico do trabalho noturno, com implicações para a vigilância em saúde do trabalhador e para políticas de prevenção do câncer na saúde coletiva.

## INTRODUÇÃO

A Agência Internacional de Pesquisa em Câncer classificou o trabalho noturno como provavelmente carcinogênico ocupacional para humanos, associado aos cânceres de mama, próstata e cólon-reto^1^. Tal classificação fundamenta-se em evidências limitadas de carcinogenicidade em humanos, combinadas a evidências suficientes provenientes de estudos experimentais e forte suporte mecanístico, que apontam a desregulação do ritmo circadiano como mecanismo biologicamente plausível para a carcinogênese^2,3^. Entretanto, os estudos epidemiológicos que investigaram a associação entre trabalho noturno e câncer ainda apresentam resultados heterogêneos e inconsistentes.

Hong et al.^4^ apontaram maior risco de câncer de mama em mulheres expostas ao trabalho noturno, enquanto Lorca et al.^5^ observaram maior risco de câncer de próstata em homens. Em contrapartida, Rivera-Izquierdo et al.^6^ e Sweeney et al.^7^ não confirmaram tais associações. Para o câncer de cólon-reto, há relatos de aumento do risco, sobretudo entre trabalhadores submetidos a turnos noturnos de longa duração^8^, embora essa associação não tenha sido observada em outros estudos^9^.

No Brasil, os estudos sobre câncer relacionado ao trabalho ainda são escassos, especialmente investigações longitudinais sobre trabalho noturno e risco de câncer. Em parte, essa limitação decorre da ausência de informações ocupacionais consistentes nas bases nacionais de câncer, dificultando a mensuração das exposições ocupacionais^10^. Além disso, a literatura epidemiológica internacional ainda apresenta limitações relacionadas à heterogeneidade na definição de trabalho noturno, à mensuração cumulativa da exposição, à temporalidade e às análises de dose-resposta. Até o momento, não foram identificados estudos prospectivos brasileiros sobre trabalho noturno e risco de câncer. Nesse contexto, o Estudo Longitudinal de Saúde do Adulto (ELSA-Brasil) constitui uma fonte estratégica para investigar essa associação, devido ao seu delineamento prospectivo e à disponibilidade de informações detalhadas sobre condições de trabalho e saúde. Adicionalmente, a caracterização da exposição ao trabalho noturno ao longo da vida ocupacional permite explorar aspectos relacionados à temporalidade e à duração da exposição.

Dessa forma, o objetivo do estudo é estimar a associação entre trabalho noturno e a incidência de câncer de mama feminino, próstata e cólon-reto na população do ELSA-Brasil, uma das maiores coortes de trabalhadores da América Latina.

## MÉTODOS

### Delineamento do Estudo

O ELSA-Brasil é uma coorte prospectiva multicêntrica iniciada em 2008, composta por mais de 15.000 servidores públicos ativos e aposentados, com idade entre 35 e 74 anos, de seis capitais brasileiras. O estudo investiga a incidência e os fatores de risco para doenças crônicas por meio de exames, entrevistas presenciais quadrienais e Entrevistas Anuais de Seguimento (EAS). Detalhes metodológicos foram descritos previamente^11,12^. Esta análise longitudinal utilizou dados da linha de base (2008–2010) e resultados sobre incidência de câncer (2008–2023).

### Participantes

Foram incluídos participantes da linha de base do ELSA-Brasil (2008–2010) livres de câncer, ativos ou aposentados, com informações completas sobre regime de trabalho, hábitos de vida e características socioeconômicas. A Figura detalha o processo de seleção da amostra, os critérios de elegibilidade e as perdas ocorridas durante o acompanhamento.

**Figura:** Fluxograma de participantes do ELSA-Brasil (2008–2023) incluídos no estudo, para cada um dos desfechos de interesse (câncer de mama, próstata e cólon-reto).

IMC: Índice de Massa Corporal; ELSA-Brasil: Estudo Longitudinal de Saúde do Adulto.

### Desfecho

Os casos incidentes de câncer de mama, próstata e cólon-reto foram identificados durante o acompanhamento da coorte (2008–2023) com base em diagnósticos médicos autorreferidos nas EAS, incluindo a localização anatômica da neoplasia. A classificação dos cânceres seguiu as diretrizes da Agência Internacional de Pesquisa em Câncer e as principais tipologias de câncer no Brasil^13^.

### Exposição

Na linha de base, a exposição ao trabalho em turnos e noturno foi mensurada por meio do questionário de História Ocupacional, com as seguintes perguntas: “Considerando todos os seus trabalhos, o(a) Sr(a) trabalha ou trabalhou em regime de plantão?” (Sim, trabalha atualmente; Sim, já trabalhou, mas não trabalha mais; Não, nunca trabalhou); “No total, considerando todos os seus trabalhos, durante quantos anos o(a) Sr(a) trabalha ou trabalhou em regime de plantão?”; “Seu regime de plantão mais frequente é ou era:” (Apenas diurno; Apenas noturno; Misto); “Por favor, descreva como é ou era seu esquema de plantão mais frequente:” (Plantão de 12/36h; Plantão de 12/60h; Um plantão de 24h semanais; Um plantão semanal de 12h; Dois plantões semanais de 12h; Outro, especifique).

Com base nos dados coletados, a variável de exposição foi definida considerando o regime de trabalho e a exposição atual e pregressa ao trabalho noturno. A classificação como trabalhador noturno seguiu os seguintes critérios: indivíduos que trabalham ou trabalharam em turnos noturnos por pelo menos 8h consecutivas entre 22h e 5h, no mínimo quatro vezes por mês. Considerou-se ainda o tempo de exposição em anos ao trabalho noturno. A definição adotada baseou-se em estudo prévio do ELSA-Brasil^14^ e buscou privilegiar a identificação de exposição noturna habitual ao longo da vida ocupacional, aumentando a especificidade da mensuração e reduzindo o potencial viés de classificação. Para avaliação da duração do trabalho noturno, os participantes foram categorizados em ≤ 10 anos e > 10 anos, com o objetivo de diferenciar exposições mais prolongadas e potencialmente associadas ao efeito cumulativo da desregulação circadiana, considerando a ausência de consenso na literatura quanto ao ponto de corte ideal para avaliação dessa exposição em estudos sobre câncer.

### Covariáveis

As covariáveis incluídas no estudo foram selecionadas com base em seu potencial de carcinogenicidade e relevância epidemiológica para as tipologias de câncer investigadas. Variáveis comportamentais reconhecidas na literatura como fatores de risco para o câncer foram: consumo excessivo de bebidas alcoólicas (consumo > 210 g/semana para homens e > 140 g/semana para mulheres); tabagismo (nunca fumou, ex-fumante, fumante atual); atividade física no lazer (fraca; moderada/forte, segundo a versão longa do questionário internacional de atividade física - IPAQ)^15^; estado nutricional avaliado pelo Índice de Massa Corporal (IMC) (magreza/eutrofia < 18 kg/m^2^ a ≤ 24,9 kg/m^2^; excesso de peso ≥ 25 kg/m^2^ a > 40 kg/m^2^); consumo de carne processada (não/sim); e consumo de carne vermelha (≤ 71 g/dia; > 71 g/dia)^16^. Variáveis sociodemográficas que podem contribuir para o desfecho de interesse incluíram: idade (variável contínua; também categorizada em 35–50 anos e > 50 anos, em razão do aumento do risco de câncer após os 50 anos na análise de incidência)^17^; sexo biológico (masculino/feminino); e escolaridade (ensino fundamental, médio, superior ou mais), escolhida em detrimento da renda. Essa decisão fundamenta-se no fato de que, embora ambos sejam indicadores socioeconômicos correlatos, a escolaridade apresenta maior estabilidade temporal, menor suscetibilidade a erro de medida e constitui um determinante estrutural da trajetória ocupacional. Assim, sua inclusão como marcador de posição socioeconômica é conceitualmente mais adequada no contexto deste estudo, conforme recomendações metodológicas de delineamentos epidemiológicos multivariados.

### Aspectos Éticos

O ELSA-Brasil foi aprovado pelos Comitês de Ética das instituições participantes e pela Comissão Nacional de Ética em Pesquisa (Conep). Este estudo foi aprovado em 26 de janeiro de 2023 pelo Comitê de Ética em Pesquisa da Escola Nacional de Saúde Pública/Fundação Oswaldo Cruz (ENSP/Fiocruz), sob o número de aprovação 66407322.3.0000.5240.

### Análise Estatística

As análises foram realizadas separadamente para cada tipo de câncer primário (mama, próstata e cólon-reto), considerando as especificidades relacionadas ao sexo. As taxas de incidência (em pessoas-ano) foram calculadas com base no tempo decorrido entre o início do seguimento e a data do diagnóstico. Os participantes que não desenvolveram os cânceres de interesse ou foram a óbito durante o período de acompanhamento foram tratados como censurados.

A associação entre trabalho noturno e incidência de câncer foi estimada por modelos de regressão de Cox, com cálculo das *hazard ratios* (HR) e intervalos de confiança de 95% (IC95%). As variáveis de ajuste foram selecionadas com base na literatura científica e nas recomendações da Agência Internacional de Pesquisa em Câncer, priorizando fatores de risco reconhecidamente carcinogênicos para as tipologias estudadas. A inclusão das covariáveis nos modelos foi feita de forma incremental, considerando ainda a significância estatística nas análises simples (p < 0,10).

Para cada uma das tipologias de câncer investigadas, foram obtidos modelos simples (modelo A). O modelo final para câncer de mama foi ajustado por consumo excessivo de álcool, tabagismo, IMC e idade (modelo B). Para câncer de próstata, o modelo final foi ajustado por consumo excessivo de álcool, tabagismo e idade (modelo D). Já o modelo final para câncer de cólon-reto foi ajustado por consumo de álcool, tabagismo, consumo de carne processada, atividade física, IMC, idade e sexo (modelo E). Adicionalmente, foi ajustado um modelo complementar com a inclusão da variável escolaridade, visando à comparação entre modelos e à verificação da ocorrência de superajuste decorrente de sua incorporação ao modelo final (modelo C).

Testes de interação entre trabalho noturno e escolaridade não indicaram modificação de efeito. A suposição de proporcionalidade dos riscos foi avaliada por meio dos gráficos de resíduos de Schoenfeld, sem evidência de violação.

As análises foram realizadas no *software* R (versão 4.4.0), com uso das bibliotecas *survival, ggsurvfit, ggplot2, ggfortify, survminer, car* e *DescTools*.

## RESULTADOS

Na linha de base do ELSA-Brasil (2008–2010), após a exclusão de participantes com diagnóstico prévio de câncer, foram incluídos 14.787 indivíduos, dos quais 81,0% trabalhavam em regime diurno. A maioria da população de estudo foi composta por mulheres (54,7%). Observou-se maior proporção de trabalho noturno entre os homens. Trabalhadores com maior escolaridade e renda concentraram-se no regime diurno, enquanto aqueles com menor escolaridade e renda foram mais frequentes no regime noturno. Entre os fatores de estilo de vida, observou-se maior prevalência de consumo excessivo de álcool, excesso de peso e menor prática de atividade física entre os plantonistas noturnos (Tabela 1).

**Tabela 1:** Distribuição das características da população de estudo, segundo regime de trabalho – ELSA-Brasil, linha de base 2008–2010. (n = 14.787)

Categoria	Regime de trabalho			Total n (%)	p-valor
	Trabalhador diurno n = 11.973 n (%)	Plantonista noturno (≤ 10 anos) n = 1.541 n (%)	Plantonista noturno (> 10 anos) n = 1.273 n (%)		
Sexo					
Masculino	5.277 (78,8)	854 (12,8)	565 (8,4)	6.696 (45,3)	< 0,0001
Feminino	6.696 (82,8)	687 (8,5)	708 (8,8)	8.091 (54,7)	
Faixa etária					
35–50 anos	5.607 (81,0)	736 (10,6)	578 (8,4)	6.921 (46,8)	0,4557
> 50 anos	6.366 (80,9)	805 (10,2)	695 (8,8)	7.866 (53,2)	
Escolaridade					
Até o ensino fundamental	1.435 (76,7)	198 (10,6)	237 (12,7)	1.870 (12,6)	< 0,0001
Ensino médio	3.878 (75,7)	601 (11,7)	645 (12,6)	5.124 (34,7)	
Ensino superior ou mais	6.660 (85,5)	742 (9,5)	391 (5,0)	7.793 (52,7)	
Renda familiar *per capita*					
Até R$ 2.879,60	3.013 (76,9)	433 (11,1)	470 (12,0)	3.916 (26,6)	< 0,0001
De R$ 2.879,70 a R$ 5.344,70	3.574 (78,7)	494 (10,9)	473 (10,4)	4.541 (30,8)	
≥ R$ 5.344,80	5.341 (85,1)	612 (9,7)	326 (5,2)	6.279 (42,6)	
Beber excessivo					
Não	11.088 (81,1)	1.397 (10,2)	1.193 (8,7)	13.678 (92,6)	0,0062
Sim	876 (79,9)	142 (12,9)	79 (7,2)	1.097 (7,4)	
Fumante					
Nunca fumou	6.861 (81,4)	842 (10,0)	726 (8,6)	8.429 (57,0)	0,3262
Ex-fumante	3.568 (80,7)	478 (10,8)	377 (8,5)	4.423 (29,9)	
Fumante	1.544 (79,8)	221 (11,4)	169 (8,7)	1.934 (13,1)	
Consumo de carne processada					
Não	1.347 (78,9)	182 (10,7)	178 (10,4)	1.707 (11,5)	0,0140
Sim	10.626 (81,2)	1.359 (10,4)	1.095 (8,4)	13.080 (88,5)	
Consumo de carne vermelha					
Moderado (≤ 71g/dia)	6.761 (82,5)	793 (9,7)	644 (7,9)	8.198 (55,4)	< 0,0001
Excessivo (> 71g/dia)	5.212 (79,1)	748 (11,4)	629 (9,5)	6.589 (44,6)	
IMC					
Magreza/eutrofia	4.512 (82,8)	542 (9,9)	394 (7,2)	5.448 (36,9)	< 0,0001
Excesso de peso	7.456 (79,9)	998 (10,7)	879 (9,4)	9.333 (63,1)	
Atividade física					
Moderada/forte	2.757 (81,7)	379 (11,2)	237 (7,0)	3.373 (23,1)	0,0006
Fraca	9.057 (80,8)	1.138 (10,2)	1.008 (9,0)	11.203 (76,9)	

IMC: Índice de Massa Corporal.

Após um acompanhamento médio de 13 anos (DP = 2,22 anos), 539 participantes (3,6%) desenvolveram as tipologias de câncer de interesse no estudo. Dentre as mulheres, 239 (3,1%) foram diagnosticadas com câncer de mama; entre os homens, 252 (4,0%) desenvolveram câncer de próstata. O câncer de cólon-reto foi identificado em 101 participantes (0,7%), sendo 51 homens e 50 mulheres. As taxas de incidência para os cânceres de mama e próstata foram de 2,34 casos (IC95% 2,05–2,66) e de 3,05 (IC95% 2,69–3,46) por 1.000 pessoas-ano, respectivamente. Já para o câncer de cólon-reto, a taxa de incidência foi de 5,63 (IC95% 4,59–6,84) por 10.000 pessoas-ano. Foram observadas maiores taxas de incidência entre indivíduos com consumo excessivo de bebidas alcoólicas para as três tipologias analisadas, alcançando o valor mais elevado para o câncer de cólon-reto (8,67 casos por 10.000 pessoas-ano). Para essa mesma tipologia, também se destacaram taxas mais altas entre aqueles que consumiam carne processada (5,72/10.000), apresentavam excesso de peso (6,03/10.000) ou relataram níveis baixos de atividade física (5,79/10.000), em comparação aos respectivos grupos de referência (Tabela 2).

**Tabela 2:** Incidência de câncer de mama, próstata e cólon-reto, segundo características sociodemográficas, comportamentais e ocupacional entre participantes do ELSA-Brasil, 2008–2023.

Variável	Câncer de mama				Câncer de próstata				Câncer de cólon-reto			
	n	Casos	Pessoa-ano	Taxa incidência^a^ IC95%	n	Casos	Pessoa-ano	Taxa incidência^a^ IC95%	n	Casos	Pessoa-ano	Taxa incidência^b^ IC95%
Sexo												
Masculino	-	-	-	-	6.333	252	82.496,8	3,05 (2,69–3,46)	6.132	51	79.819,8	6,39 (4,76–8,40)
Feminino	7.611	239	102.133,8	2,34 (2,05–2,66)	-	-	-	-	7.422	50	99.580,6	5,02 (3,73–6,62)
Faixa etária												
35–50 anos	3.613	103	49.402,5	2,08 (1,70–2,53)	3.076	36	41.344,7	0,87 (0,61–1,21)	6.575	25	89.212,8	2,80 (1,81–4,14)
> 50 anos	3.998	136	52.731,4	2,58 (2,16–3,05)	3.257	216	41.152,1	5,25 (4,57–6,00)	6.979	76	90.187,6	8,43 (6,64–10,55)
Escolaridade												
Fundamental ou menos	737	16	9.396,5	1,70 (0,97–2,77)	1.075	37	13.316,9	2,78 (1,96–3,83)	1.770	11	22.155,5	4,96 (2,48–8,88)
Médio	2.798	61	37.707,1	1,62 (1,24–2,08)	2.144	52	28.134,8	1,85 (1,38–2,42)	4.861	32	64.725,6	4,94 (3,38–6,98)
Superior ou mais	4.076	162	55.030,2	2,94 (2,51–3,43)	3.114	163	41.045,1	3,97 (3,38–4,63)	6.923	58	92.519,4	6,27 (4,76–8,10)
Renda												
Até R$ 2.489,00	2.066	36	27.533,4	1,31 (0,92–1,81)	1.727	55	22.258,8	2,47 (1,86–3,22)	3.723	21	48.847,7	4,30 (2,66–6,57)
De R$ 2.490,00 a R$ 4.979,00	2.482	74	33.423,9	2,21 (1,74–2,78)	1.869	47	24.393,6	1,93 (1,42–2,56)	4.260	30	56.556,4	5,30 (3,58–7,57)
≥ R$ 4.980,00	3.027	129	40.765,3	3,16 (2,64–3,76)	2.708	148	35.523,8	4,17 (3,52–4,89)	5.508	50	73.291,5	6,82 (5,06–8,99)
Consumo excessivo de bebidas alcoólicas												
Não	7.330	225	98.463,0	2,29 (2,00–2,60)	5.565	220	72.765,8	3,02 (2,64–3,45)	12.540	90	166.468,3	5,41 (4,35–6,65)
Sim	263	14	3.485,8	4,02 (2,20–6,74)	760	32	9.674,6	3,31 (2,26–4,67)	988	11	12.690,7	8,67 (4,33–15,51)
Tabagismo												
Nunca fumou	4.753	155	64.140,0	2,42 (2,05–2,83)	3.211	118	42.522,8	2,77 (2,30–3,32)	7.742	51	103.659,0	4,92 (3,66–6,47)
Ex-fumante	1.923	61	25.678,4	2,38 (1,82–3,05)	2.224	107	28.745,1	3,72 (3,05–4,50)	4.011	32	52.598,6	6,08 (4,16–8,59)
Fumante	935	23	12.315,5	1,87 (1,18–2,80)	897	27	11.214,8	2,41 (1,59–3,50)	7.742	51	103.659,0	4,92 (3,66–6,47)
Consumo de carne processada												
Não	996	29	13.047,3	2,22 (1,49–3,19)	625	37	7.939,4	4,66 (3,28–6,42)	1.565	10	20.237,6	4,94 (2,37–9,09)
Sim	6.615	210	89.086,5	2,36 (2,05–2,70)	5.708	215	74.557,5	2,88 (2,51–3,30)	11.989	91	159.162,9	5,72 (4,60–7,02)
Consumo de carne vermelha												
≤ 71g/dia	4.820	167	64.630,9	2,58 (2,21–3,01)	2.885	119	37.485,4	3,17 (2,63–3,80)	7.470	51	98.970,2	5,15 (3,84–6,78)
> 71g/dia	2.791	72	37.503,0	1,92 (1,50–2,42)	3.448	133	45.011,4	2,95 (2,47–3,50)	6.084	50	80.430,3	6,22 (4,61–8,20)
IMC												
Magreza/eutrofia	2.997	89	40.424,6	2,20 (1,77–2,71)	2.152	92	28.202,6	3,26 (2,63–4,00)	5.001	33	66.611,0	4,95 (3,41–6,96)
Excesso de peso	4.611	150	61.674,9	2,43 (2,06–2,85)	4.179	160	54.266,5	2,95 (2,51–3,44)	8.548	68	112.727,4	6,03 (4,68–7,65)
Atividade física												
Moderada/forte	1.478	50	19.940,6	2,51 (1,86–3,31)	1.649	90	21.675,6	4,15 (3,34–5,10)	3.009	22	40.050,0	5,49 (3,44–8,32)
Fraca	6.019	189	80.579,6	2,35 (2,02–2,70)	4.592	159	59.505,8	2,67 (2,27–3,12)	10.342	79	136.465,9	5,79 (4,58–7,21)
Regime de trabalho												
Trabalhador diurno	6.193	200	83.045,0	2,41 (2,09–2,77)	4.856	197	63.370,8	3,11 (2,69–3,57)	10.729	77	142.135,4	5,42 (4,28–6,77)
Plantonista noturno ≤ 10 anos	642	24	8.600,2	2,79 (1,79–4,15)	778	25	10.142,5	2,46 (1,60–3,64)	1.385	14	18.273,3	7,66 (4,19–12,85)
Plantonista noturno >10 anos	665	11	9.044,8	1,22 (0,61–2,18)	533	25	6.842,2	3,65 (2,36–5,39)	1.169	7	15.484,6	4,52 (1,82–9,31)

IC95%: intervalo de confiança de 95%.

^a^ Taxas de evento por 1.000 pessoas-ano.

^b^ Taxas de evento por 10.000 pessoas-ano.

Considerando o trabalho noturno e as três tipologias de câncer avaliadas, não foi observada associação com o câncer de mama entre as mulheres (HR = 0,89; IC95% 0,65–1,22). Já em relação ao câncer de próstata, após o ajuste para consumo de bebida alcoólica, tabagismo e idade, os homens expostos ao trabalho noturno por mais de 10 anos tiveram um aumento de 38% no risco de câncer para esta tipologia (IC95% 1,09–1,75). A exposição ao trabalho noturno por mais de 10 anos, após ajuste por consumo de bebida alcoólica, tabagismo, carne processada, atividade física no lazer, IMC, idade e sexo, esteve associada a um aumento no risco de desenvolver câncer de cólon-reto (HR = 1,35; IC95% 1,09–1,68). Contudo, a força da associação mencionada, tanto para câncer de próstata quanto para cólon-reto, foi atenuada pelo ajuste por escolaridade (Tabela 3).

**Tabela 3:** Risco de câncer de mama, próstata e cólon-reto, segundo exposição ao trabalho noturno entre participantes do ELSA-Brasil 2008–2023.

Categoria de exposição	Câncer de mama			Câncer de próstata			Câncer de cólon-reto		
	HR (IC95%)^a^	HR (IC95%)^b^	HR (IC95%)^c^	HR (IC95%)^a^	HR (IC95%)^d^	HR (IC95%)^c^	HR (IC95%)^a^	HR (IC95%)^e^	HR (IC95%)^c^
Trabalho diurno (Referência)	1,00	1,00	1,00	1,00	1,00	1,00	1,00	1,00	1,00
Trabalho noturno ≤ 10 anos	0,98 (0,72–1,34)	1,00 (0,73–1,37)	1,00 (0,73–1,37)	1,00 (0,79–1,26)	1,05 (0,83–1,32)	0,99 (0,78–1,24)	1,13 (0,91–1,41)	1,07 (0,86–1,34)	0,99 (0,79–1,24)
Trabalho noturno >10 anos	0,92 (0,67–1,25)	0,89 (0,65–1,22)	0,87 (0,64–1,20)	1,41 (1,12–1,79)	1,38 (1,09–1,75)	1,24 (0,98–1,57)	1,38 (1,11–1,71)	1,35 (1,09–1,68)	1,15 (0,92–1,43)

HR: *hazard ratio*; IC95%: intervalo de confiança de 95%.

^a^ Modelo bruto de regressão de Cox.

^b^ Modelo de regressão de Cox ajustado por: bebida alcoólica, tabagismo, IMC e idade.

^c^ Ajuste adicional por escolaridade.

^d^ Modelo de regressão de Cox ajustado por: bebida alcoólica, tabagismo e idade.

^e^ Modelo de regressão de Cox ajustado por: bebida alcoólica, tabagismo, carne processada, atividade física, IMC, sexo e idade.

## DISCUSSÃO

O objetivo deste estudo foi estimar a associação entre trabalho noturno e incidência de câncer de mama feminino, próstata e cólon-reto na população do ELSA-Brasil. Nessa grande coorte de servidores públicos brasileiros, a exposição ao trabalho noturno por mais de 10 anos foi associada ao aumento do risco de câncer de próstata e colorretal, em comparação a trabalhadores diurnos e àqueles com menor tempo de exposição.

Nossos resultados estão em consonância com evidências de coortes nos Estados Unidos, com mulheres^18^, e na Noruega, com homens^19^, que identificaram aumento de 64% no risco de câncer colorretal e de 86% no risco de câncer de próstata, respectivamente. Ambos consideraram o tempo de trabalho em turnos noturnos a partir de 10 anos. Um ponto comum e relevante entre tais investigações e o presente estudo é que as análises consideraram a exposição ao trabalho noturno ao longo da vida, e não apenas a exposição atual. Isso permitiu observar com mais precisão os efeitos acumulados do trabalho noturno no desenvolvimento do câncer por meio do histórico ocupacional.

Por outro lado, estudos não longitudinais^20^, com seguimento médio inferior a 10 anos^21^, ou que mensuraram a exposição a partir do horário de trabalho atual no início do seguimento^22^ relataram ausência de associação entre o trabalho noturno e o risco de câncer de cólon-reto e de próstata. Acreditamos que o efeito do trabalho noturno no desenvolvimento de câncer, que geralmente possui um longo período de latência, especialmente para tumores sólidos, pode ser adequadamente avaliado em estudos de longo prazo. Além disso, é essencial incluir o levantamento completo do histórico ocupacional dos trabalhadores, o que possibilita contabilizar a exposição pregressa, incluindo sua duração, reduzindo, assim, as chances de estimativas de risco subestimadas.

Esse entendimento é corroborado por autores como Harma et al.^23^, que sugerem que um tempo de acompanhamento reduzido e a ausência de informações sobre a exposição anterior à linha de base, limitam a capacidade de estudos de coorte em avaliar os efeitos de longo prazo do trabalho noturno sobre a saúde. Shi et al.^8^ observaram que o risco de câncer colorretal aumentou significativamente com a exposição ao trabalho noturno rotativo de longa duração, especialmente em trabalhadores expostos por 15 anos ou mais.

Adicionalmente, Gustavsson et al.^24^ relataram um aumento no risco de câncer de mama (HR = 4,33; IC95% 1,45–10,57), entre mulheres que trabalharam à noite por oito anos ou mais, em uma coorte sueca. No entanto, os autores concluíram que as limitações de seu estudo, como o curto tempo de acompanhamento, o baixo número de casos de trabalhadores com exposição prolongada ao trabalho noturno e a falta de informações sobre o histórico ocupacional antes da linha de base, comprometeram a precisão das estimativas. Esses resultados reforçam a importância de investigações longitudinais, entre trabalhadores expostos de forma prolongada, para que se possa avaliar o real impacto da exposição ao trabalho noturno no desenvolvimento de neoplasias mamárias.

Dessa forma, nossa hipótese é que a ausência de associação observada nas primeiras estimativas de risco para o câncer de mama em nossa coorte brasileira (ELSA-Brasil) possa estar relacionada à necessidade de um acompanhamento mais prolongado, que considere um quantitativo maior de trabalhadores expostos, bem como o longo período de latência característico dessa neoplasia.

Em relação à atenuação das estimativas após o ajuste dos modelos de próstata e cólon-reto pela variável escolaridade, reafirmamos que fatores socioeconômicos exercem forte influência no adoecimento e na mortalidade por câncer no contexto brasileiro^25^. Entretanto, considerando as especificidades deste estudo, que busca contribuir para a compreensão da complexa relação entre câncer e trabalho em uma coorte de trabalhadores brasileiros, não é possível analisar a escolaridade de forma isolada, como um fator socioeconômico que atuaria apenas como confundidor, uma vez que ela integra a dimensão sócio-ocupacional que condiciona a probabilidade de exposição ao trabalho noturno. Em outras palavras, a exposição ao trabalho noturno depende do tipo de ocupação, que, por sua vez, é profundamente influenciado pelo nível de escolaridade do indivíduo. No presente estudo, observou-se que 69,28% dos trabalhadores expostos ao trabalho noturno por mais de 10 anos possuíam até o ensino médio (Tabela 1), evidenciando que a permanência prolongada nesse regime tende a ocorrer entre indivíduos com menor escolaridade.

Nesse contexto, levantamos a hipótese de que trabalhadores com maior qualificação tendem a possuir maior autonomia para reorganizar sua trajetória profissional e, sempre que possível, migram para jornadas diurnas, reduzindo o tempo total de exposição ao turno noturno. Já aqueles com menor escolaridade, cuja inserção ocupacional costuma ser mais restrita, frequentemente dispõem de menos alternativas para alterar seus horários de trabalho, o que contribui para exposições mais longas e persistentes ao trabalho noturno. Essa dinâmica é coerente com a literatura que descreve a forte estratificação socioeconômica na distribuição das condições de trabalho e das possibilidades de mobilidade ocupacional^26^, reforçando a necessidade de considerar a escolaridade como parte integrante da estrutura que determina a própria exposição, e não apenas como um possível confundidor.

Portanto, no contexto deste estudo, incluir a escolaridade no modelo final configuraria um superajuste. A literatura epidemiológica enfatiza que o superajuste dilui associações reais, aumenta o erro-padrão e compromete a interpretação causal^27^. No caso da exposição ao trabalho noturno, a escolaridade integra os correlatos estruturais da exposição e, portanto, não deve ser incluída automaticamente nos modelos^27^. Ao controlar variáveis que compõem a própria estrutura geradora da exposição, corre-se o risco de reduzir artificialmente a magnitude do efeito e subestimar o risco verdadeiro. Assim, optamos metodologicamente por não ajustar pela escolaridade, a fim de preservar a validade interna das estimativas e evitar vieses decorrentes de superajuste, especialmente em um campo no qual ainda há escassez de estudos longitudinais nacionais capazes de elucidar a relação entre trabalho noturno e a incidência de câncer.

À luz dessa justificativa metodológica, os achados deste estudo ganham maior consistência, uma vez que a ausência de ajuste por variáveis estruturalmente vinculadas à exposição permite captar de forma mais fidedigna seus efeitos potenciais. Nossos resultados apontam para a existência de uma associação entre o trabalho noturno de longa duração e o câncer, tendo em vista que a exposição crônica a essa condição pode favorecer o desenvolvimento da doença por meio de mecanismos como a indução de processos inflamatórios e a desregulação do ciclo circadiano, corroborando os achados de outros autores.

De acordo com a Agência Internacional de Pesquisa em Câncer^1^, a exposição à luz artificial durante o trabalho noturno é a principal razão para a desregulação do ritmo circadiano. Li et al.^3^ destacam que esse fenômeno é considerado um possível mecanismo que favorece o surgimento de câncer por prejudicar o controle dos ciclos celulares, reduzir a eficiência da resposta imunológica e do reparo do DNA, com implicações diretas na gênese do câncer. Autores enfatizam que a associação entre a exposição ao trabalho noturno e o desenvolvimento de cânceres endócrinos, como os de mama, próstata e cólon-reto, é sustentada por fortes evidências mecanicistas e estudos experimentais com animais. Esses estudos revelam o papel crucial dos genes do relógio circadiano na regulação de processos hormonais, além de influenciar a secreção de enzimas, a absorção de fluidos e nutrientes. A exposição prolongada ao trabalho noturno causa mudanças substanciais nos ritmos fisiológicos, impactando negativamente o equilíbrio hormonal e metabólico, o que pode aumentar o risco de câncer^28,29^. Tais evidências reforçam a relevância de considerar os impactos da desregulação circadiana na saúde humana, sobretudo em trabalhadores expostos cronicamente a turnos noturnos. No entanto, as relações entre o trabalho noturno e o risco de câncer em estudos epidemiológicos ainda não estão plenamente estabelecidas. Nesse sentido, nosso estudo contribui para preencher essa lacuna, especialmente no contexto brasileiro, onde são escassas as investigações longitudinais capazes de avaliar de forma prospectiva os efeitos do trabalho noturno sobre a incidência de câncer.

Por fim, destacamos a relevância deste estudo como a primeira coorte de trabalhadores a estimar o risco de câncer associado ao trabalho noturno no Brasil, representando um avanço inédito no campo da saúde do trabalhador. A robustez do ELSA-Brasil, com ampla amostra e informações detalhadas sobre as exposições, confere solidez e originalidade ao nosso estudo.

Apesar dos pontos fortes, os achados devem ser interpretados à luz de limitações inerentes aos estudos epidemiológicos ocupacionais. Destaca-se a possibilidade de ocorrência do *healthy worker effect*, fenômeno em que trabalhadores ativos tendem a apresentar melhores condições de saúde que a população geral, podendo contribuir para a subestimação do risco associado ao trabalho noturno. Além disso, salienta-se a necessidade de um período de acompanhamento mais extenso, considerando o longo período de latência dos tumores sólidos. O quantitativo reduzido de trabalhadores expostos e, consequentemente, o baixo número de casos ao considerar a duração da exposição ao trabalho noturno também podem limitar a avaliação de um possível gradiente de risco, o que poderia evidenciar de forma mais consistente a associação entre o tempo de exposição e o desenvolvimento dos cânceres investigados, especialmente o câncer de mama.

Cabe ainda mencionar que os casos incidentes de câncer foram identificados por diagnósticos médicos autorreferidos nas EAS, sem validação por registros clínicos ou laudos médicos, podendo ocasionar viés de classificação. Além disso, a exposição ao trabalho noturno e as covariáveis relacionadas ao estilo de vida foram autorreferidas e mensuradas apenas na linha de base, podendo reduzir a precisão das medidas ao longo do seguimento e contribuir para a subestimação das associações observadas.

## CONCLUSÃO

Nossos resultados sugerem que a exposição prolongada ao trabalho noturno habitual pode estar associada ao aumento do risco de câncer, especialmente de próstata e colorretal. Nesse contexto, o tempo de exposição emerge como aspecto relevante na avaliação desse risco, sustentando a hipótese de possível efeito cumulativo e de relação dose-resposta entre trabalho noturno e carcinogênese. Esses achados ampliam o entendimento sobre a influência do ambiente laboral na saúde de servidores públicos brasileiros, ratificando o potencial carcinogênico das atividades realizadas no período da noite. O estudo reforça a importância de discutir os riscos ocupacionais e implementar políticas preventivas e intervenções de saúde pública voltadas ao trabalho noturno.
